# Differential Evolution of α-Glucan Water Dikinase (GWD) in Plants

**DOI:** 10.3390/plants9091101

**Published:** 2020-08-27

**Authors:** Muyiwa S. Adegbaju, Olanrewaju B. Morenikeji, Eli J. Borrego, André O. Hudson, Bolaji N. Thomas

**Affiliations:** 1 Institute for Plant Biotechnology, Stellenbosch University, Stellenbosch 7600, South Africa; 20563124@sun.ac.za; 2 Department of Biomedical Sciences, College of Health Science and Technology, Rochester Institute of Technology, Rochester, NY 14623, USA; omorenik@hamilton.edu; 3 Department of Biology, Hamilton College, Clinton, NY 14623, USA; 4 Thomas H. Gosnell School of Life Sciences, Rochester Institute of Technology, Rochester, NY 14623, USA; ejbsbi@rit.edu (E.J.B.); aohsbi@rit.edu (A.O.H.)

**Keywords:** GWD, starch phosphorylation, enzyme, evolution, plants

## Abstract

The alpha-glucan water dikinase (GWD) enzyme catalyzes starch phosphorylation, an integral step in transitory starch degradation. The high phosphate content in stored starch has great industrial value, due to its physio–chemical properties making it more versatile, although the phosphate content of stored starch varies depending on the botanical source. In this study, we used various computational approaches to gain insights into the evolution of the GWD protein in 48 plant species with possible roles in enzyme function and alteration of phosphate content in their stored starch. Our analyses identified deleterious mutations, particularly in the highly conserved 5 aromatic amino acid residues in the dual tandem carbohydrate binding modules (CBM-45) of GWD protein in *C. zofingiensis*, *G. hirsutum*, *A. protothecoides*, *P. miliaceum*, and *C. reinhardtii.* These findings will inform experimental designs for simultaneous repression of genes coding for GWD and the predicted interacting proteins to elucidate the role this enzyme plays in starch degradation. Our results reveal significant diversity in the evolution of GWD enzyme across plant species, which may be evolutionarily advantageous according to the varying needs for phosphorylated stored starch between plants and environments.

## 1. Introduction

The plastid-localized alpha-glucan water dikinase (*GWD*) gene is encoded by the nuclear genome and its products catalyzes reactions responsible for starch phosphorylation, an essential step in the de novo biosynthesis of this polysaccharide [1]. Generally, two plastidial isoforms of GWD; GWD1 (EC 2.7.9.4) and phosphoglucan water dikinase (PWD) or GWD3 (PWD; EC 2.7.9.5) have been identified in most plants [2]. The dikinase mechanism of GWD and PWD involve autophosphorylation of the catalytic histidine by the β-phosphate of ATP, and the transfer of β-phosphate from the stable phosphohistidine to either the C3 or C6 position of the glucosyl residue of starch, while the *γ*-phosphate is transferred to water [1,3]. Glucosyl residues in its C6 positions are phosphorylated by GWD1 [4] while pre-phosphorylated starch is further phosphorylated in its C3 positions by PWD [5,6]. Strong experimental evidence demonstrates that PWD acts downstream of GWD1 because repression of GWD1 activity leads to elimination of PWD activity [7]. Furthermore, GWD1 and PWD have been linked to starch degradation. While less is known about this phenomenon, the model for plastidial starch degradation starts with its phosphorylation, orchestrated by the activities of GWD1 and PWD [2]. In addition to the pervasive GWD isoforms, another isoform, GWD2, uniquely localized in the cytosol, has been identified [8]. Mainly because of its cytosolic localization, GWD2 has not been associated with transitory starch degradation [8].

GWD’s importance is not limited to its role in the biosynthesis or degradation of starch, but also accounts for starch-bound phosphate (SBP) in industrial starches. High phosphate content is desirable in industrial starches because it prevents or minimizes the need for artificial increase of phosphate starch content, with costly chemicals that are less-friendly to the environment. Nevertheless, an artificial increase in starch phosphate is often necessary because SBP is species and tissue type-specific in planta. For example, *Zea mays* seeds from which most industrial starch is extracted have negligible SBP [9], whereas 0.1–0.5% of glucose residues are phosphorylated in *Solanum tuberosum* [10]. Biotechnological processes provide safer alternatives to artificial augmentation of starch phosphate, and their application have yielded tremendous results [11]. Overexpression of *S. tuberosum* GWD led to double the amount of SBP in *Manihot esculenta* [12], while a six-fold increase was reported in *S. tuberosum* when the two isoforms of the starch branching enzyme were simultaneously repressed [13], including an increase in SBP when engineered laforin (a human enzyme) was expressed by potato [11].

Therefore, varying levels of phosphorylated starch prompts the hypothesis that GWD has undergone differentiation in different plant species, thereby altering its catalytic activity or substrate binding capacity. Enzymatic evolution, allow organisms to cope with changing and adverse environmental conditions, by providing novel molecular machinery capable of diversifying biochemical reactions [14]. In fact, Thalman and Santelia [15], opined that plants mobilize starch to cope during abiotic stress, strengthening the argument for the species-based evolution of GWD during contrary environmental pressures. Such evolution occurs via incremental residue mutation; insertion or deletion (indel) of amino acid residues; gene fusion and fission; protein oligomerization and post-transitional modification [16], which often results in substrate and functional selectivity. It may also alter binding specificity between enzyme and its substrate, this contingent on the degree of structural modification [17]. Variants of some of the reported proteins (with the same tertiary structure and performing similar biological functions but with different amino acid sequences) have been linked to the evolutionary divergence of species [18]. However, amino acid residues needed for maintaining protein 3D structure and biological activity are usually conserved while amino acid substitutions are common in positions that are less critical for function. Orthologs may retain biological function and structure across species, while modifications in other species [19,20] may have resulted in loss [21,22] or gain of functions [23,24,25].

The characterization of specific enzymatic functions is time and resource-intensive [26]. There is a rising number of gene and protein sequences available in public databases, enabling rapid and economically feasible evolutionary and functional prediction studies [16]. In this study, we utilized a comprehensive computational approach to understand GWD protein sequence diversity in selected agronomical and model plant species, and its contribution to substrate specificity and function. The knowledge generated will expedite functional studies into the biology and biochemistry of starch phosphorylation and guide gene editing approaches to potentially generate high phosphate starch in agro–economically important crop species.

## 2. Materials and Methods

### 2.1. Sequence Curation and Multiple Sequence Alignment

We studied the molecular evolution of the GWD enzyme in the following plant divisions (phyla); rhodophytes, chlorophytes, lycophytes, bryophytes and magnoliophytes (angiosperms). GWD protein sequences were curated from the following databases; UniProtKB/Swiss-Prot, GenBank, and Phytozome. Their designated identities/accession numbers are shown on Supplementary Table S1 (Supplementary Materials) A total of 48 plants were selected: *Porphyra umbilicalis*, Irish moss (*Chondrus crispus*), *Chromochloris zofingiensis*, Green microalgae (*Auxenochlorella protothecoides*), *Chlamydomonas reinhardtii*, *Selaginella moellendorff* Liverwort (*Marchantia polymorpha*), *Sphagnum magellanicum*, Moss (*Physcomitrella patens*), *Amborella trichopoda*, *Linum usitatissimum*, Cassava (*Manihot esculenta*), Castor bean (*Ricinus communis*), Cotton (*Gossypium hirsutum*), Cocoa (*Theobroma cacao*), *Citrus clementina*, *Malcolmia maritima*, *Capsella rubella*, *Arabidopsis thaliana*, *Myagrum perfoliatum*, Cabbage (*Brassica oleracea capitate*), *Brassica rapa*, White yam (*Dioscorea alata*), Broom millet (*Panicum miliaceum*), Banana (*Musa acumulata subsp malaccensis*), Pineapple (*Ananas comosus*), *Sorghum bicolor*, Maize (*Zea mays*), Rice (*Oryza sativa Japonica group*), *Brachypodium distachyon,* Barley (*Hordeum vulgare*), Wheat (*Triticum aestivum*), *Amaranthus hypochondriacus*, Common sunflower (*Helianthus annuus*), Coffee (*Coffea arabica*), Tobacco (*Nicotiana tabacum*), Pepper (*Capsicum annuum*),Tomato (*Solanum lycopersicum*),Chaco potato (*Solanum chacoense*), Potato (*Solanum tuberosum*), Grape (*Vitis vinifera*), Strawberry (*Fragaria vesca*), Apple (*Malus domestica*), Muskmelon (*Cucumis melo*), Soybean (*Glycine max*), *Phaseolus vulgaris*, *Vigna unguiculata*, Pawpaw (*Carica papaya*).

All the GWD protein sequences were compiled into a single file in Jalview 2.11.0 [27], and saved in a FASTA format. We computed multiple sequence alignment on the phylogenetic platform [28], utilizing Multiple Alignment using the Fast Fourier Transform (MAFFT) [29], with the following settings: gap extend penalty (0.123); gap opening penalty (1.53). Accuracy of phylogenetic inference was enhanced by block mapping and gathering with entropy (BMGE) [30]. BMGE implements trimming to reduce artefacts, therefore, only phylogenetic informative regions from such trimmed outputs were used to reconstruct the phylogenetic tree. Maximum likelihood phylogenetic tree PhyML [31] was generated with the following settings; the model selection option is Smart Model Selection (SMS) method [32] and AIC scores. Node support was also determined using approximate likelihood ratio test with Shimodaira–Hasegawa-like estimate (SH-like aLRT) [33]. The phylogenetic tree was formatted using interactive Tree Of Life (iTOL) [34].

### 2.2. Comparative Analysis of GWD Protein Primary Structure

The physical and chemical properties of GWD orthologs were analyzed with ProtParam [35]. The following properties were calculated: aliphatic index, instability index, molecular weight and grand average of hydropathicity (GRAVY), as described [36]. Cysteine count in each of the GWD sequences and disulfide connectivity predictions was done according to the method of Ferre and Clote [37,38].

### 2.3. Prediction of Signal Sequence, Motif Scanning and Analysis of Domains

The N-terminal region of the multiple sequence alignment (MSA) of all the GWD amino acid sequences was scanned for the presence of carbohydrate binding module (CBM). Using the potato GWD sequence as reference, the two tandems of CBM45 were extracted from the MSA with Snapgene version 4.3.8 (San Diego, CA, United States of America) and formatted by Corel X8. The MSA was also scanned for redox-regulation motif (CFATC) as reported [39]. Prediction of the presence of transit signal peptides, subcellular localization and cleavage site was carried out using TargetP-2 [40]. Some of the result of TargetP-2 was compared with those obtained from Multiloc2, which uses MultiLoc2-LowRes (Plant), 5 localizations prediction methods [41].

### 2.4. Amino acid Substitution Prediction

To test the effect of substitution and deletion among the conserved five aromatic amino acids in the two CBM45 modules and the redox-regulation motif, we used Protein Variation Effect Analyser (PROVEAN) to predict mutation effects [42]. The potato GWD sequence was used as the query sequence and effects of point mutations in those positions were predicted. The substitution or deletion effect was scored deleterious (capable of altering enzyme function) if the score was 2.5 or higher or scored neutral (enzyme still able to perform its function) if it was lesser than 2.5.

## 3. Results

### 3.1. Sequence Comparison and Developmental Pattern

**Figure 1:**
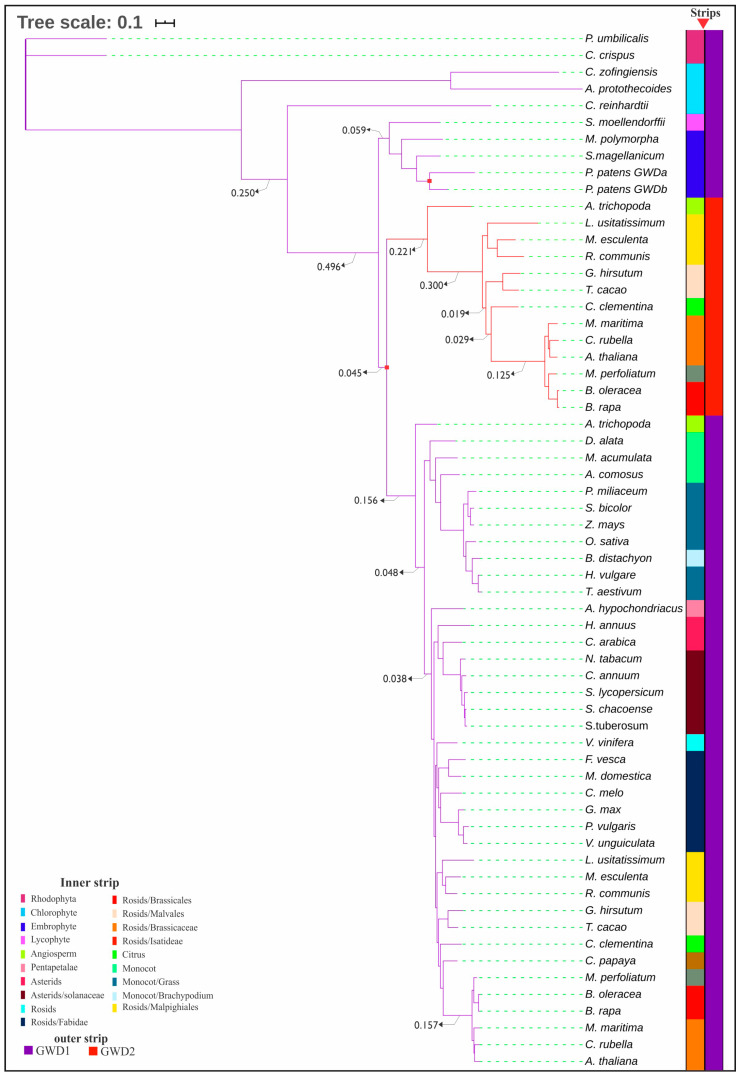
Evolutionary relationship among the taxa as shown by the phylogenetic tree. We used block mapping and gathering with entropy (BMGE) for trimming MSA of GWD protein sequences. For tree inference (PhyML + SMS), default setting for statistical model, tree topology search and branch support was used. The scale represents amino acid substitution per site. Duplication of ancestral alpha-glucan water dikinase (GWD) in *P. patens* to GWDa and GWDb, as well as GWD1 and GWD2 in angiosperms are indicated with red squares at the respective nodes.

GWD transcripts were found in all the plant phyla examined, i.e., rhodophytes, chlorophytes, lycophytes, bryophytes and angiosperms. The phylogenetic tree grouped all the plants in their respective phyla, angiosperms branched further into asterids and rosids. For instance, in Fabidae (a rosid family), *F. vesca*, *M. domestica*, *C. melo*, *G. max*, *P. vulgaris* and *V. unguiculata* were all grouped in the same clade. Our result showed that GWD enzyme in rhodophytes (*P. umbilicalis and C. crispus*) was most distant from the rest of plant phyla, forming an out-group (Figure 1). The primary structure of GWD protein in rhodophytes may have been the closest to the ancestral GWD protein of other phyla due to shortness of its branch and absence of diverged clade. Conversely, *A. protothecoides* a chlorophyte may have diverged the most from ancestral protein of GWD, going by its branch length, followed by another chlorophyte; *C. zofingiensis*. Two highly similar GWD sequences were found for *P. patens*—GWDa and GWDb. GWD duplication, which occupies a distinctive clade and referred to as GWD2 occurred in *A. trichopoda* and the dicot families; malpighiales, malvales, citrus, brassicales and brassicaceae, but was not found in monocots or other dicots. The average branches from the root of GWD2 clade and its sub-clades are longer and more varied when compared with those of GWD1 in angiosperm group. However, slight sequence divergence existed between GWD1 of the angiosperm group and its paralog GWD2, as indicated by their close proximities to the root of their clades.

### 3.2. Assessment of Physio–Chemical Properties of GWD Protein Sequences

We examined the primary structure of GWD protein sequences for the plants under study to elucidate its function (Table 1). We report the average length of GWD1 to be 1422 aa while that of GWD2 is ~1285 aa. The aliphatic index of GWD protein was moderately high, regardless of the protein subgroup, indicating moderate protein thermostability and solubility. The in vivo half-life of GWD1, measured by instability index, revealed a longer half-life (instability index < 40) in *A. protothecoides* (34.80), *T. aestivum* (37.49), *P. patens* GWDb (37.61), *P. umbilicalis* (38.61)*, C. reinhardtii* (39.07), *C. crispus* (39.12), *V. vinifera* (39.46) and *S. moellendorffi* (39.89), with similar observation for GWD2 in *T. cacao* (38.25), *M. perfoliatum* (38.33)*, M. esculenta* (38.41) *and B. rapa* (38.83). The higher instability indexes (>40) in the other plants indicate a shorter half-life. *C. reinhardtii* had 24 cysteines in its GWD1 sequence (the highest among all), followed by *C. crispus* and *M. acumulata* (15), *A. comosus* and *P. patens* GWDb (14). The number of cysteines in GWD2 subgroup however was unusually higher among plants with this paralog (doubled for most of the plants and nearly tripled in *B. oleracea*-31). Furthermore, though the GWD1 of rhodophytes, chlorophytes and bryophytes had higher cysteine composition than angiosperms, some fluctuation was recorded; slight difference among rhodophytes; *P. umbilicalis* (18) *and C. crispus* (15), gave way to wide variation among chlorophytes; *C. zofingiensis* (18), *A. protothecoides* (10) *and C. reinhardtii* (24), with bryophytes following a similar trend.

### 3.3. Prediction of Transit Peptide, Motif and Analysis of Functional Domain

We scanned the GWD1 and GWD2 peptide sequences for presence of organelle-targeting signal sequences, expected to reside at the N-terminus. Our result with TargetP-2 show that signal sequences exist in the GWD1 of most of the plants (Table 2); *C. reinhartii*, all monocots except *M. acumulata* and *H. vulgare*, solanecea, malpighiales, malvales, citrus, brassicales and all brassicaceae except *M. maritima*, predicted to be targeting chloroplast. However, no signal sequence was identified for any of the GWD2. Additionally, our analysis predicted signal sequence for *P. patens* GWDb, *H. vulgare* and *M. maritima* to be mitochondrial transit peptide (mTP). Furthermore, the predicted length of the signal sequence varied, with *D. alata* possessing the longest (between 96–97 amino acids) and *H. vulgare* with the shortest (16–17). 

### 3.4. Carbohydrate Binding Module (CBM45) Tandem Domains

**Figure 2:**
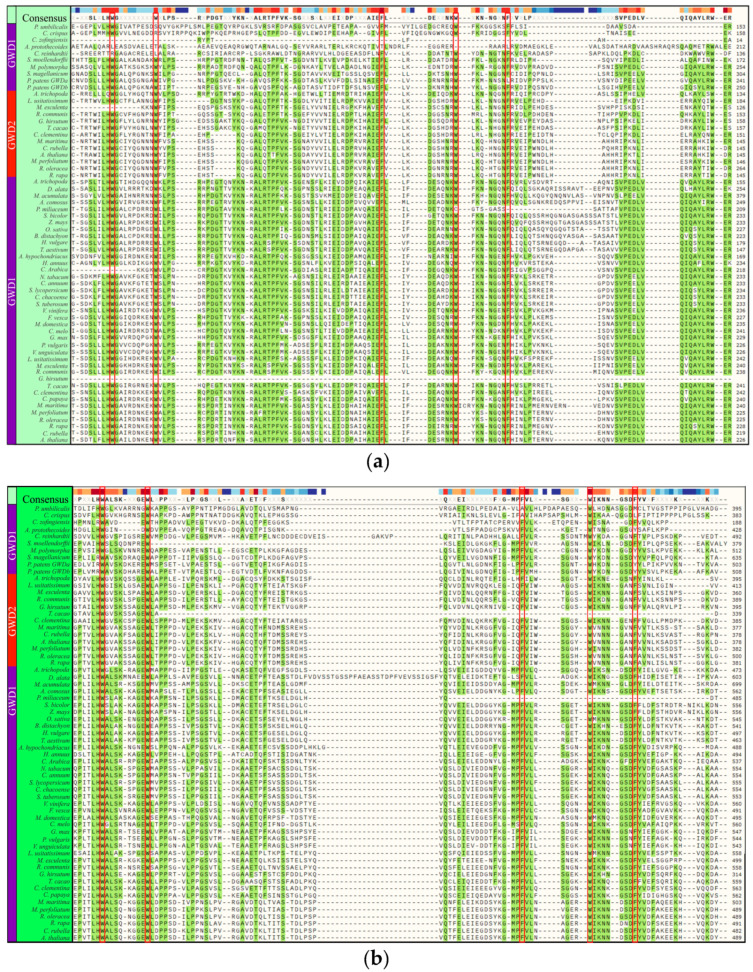
(**a**) Multiple sequence alignment of the first tandem carbohydrate binding module (CBM45-1) showing the five consensus aromatic residues (W, W, F, W, F, respectively) associated with starch binding. The five conserved aromatic amino acids in the (CBM45-1) are highlighted in red. (**b**) Multiple sequence alignment of the second tandem carbohydrate binding module (CBM45-2) showing the five consensus aromatic residues (W, W, F, W, F, respectively) associated with starch binding. The five conserved aromatic amino acids in the (CBM45-2) are highlighted in red.

**Figure 3:**
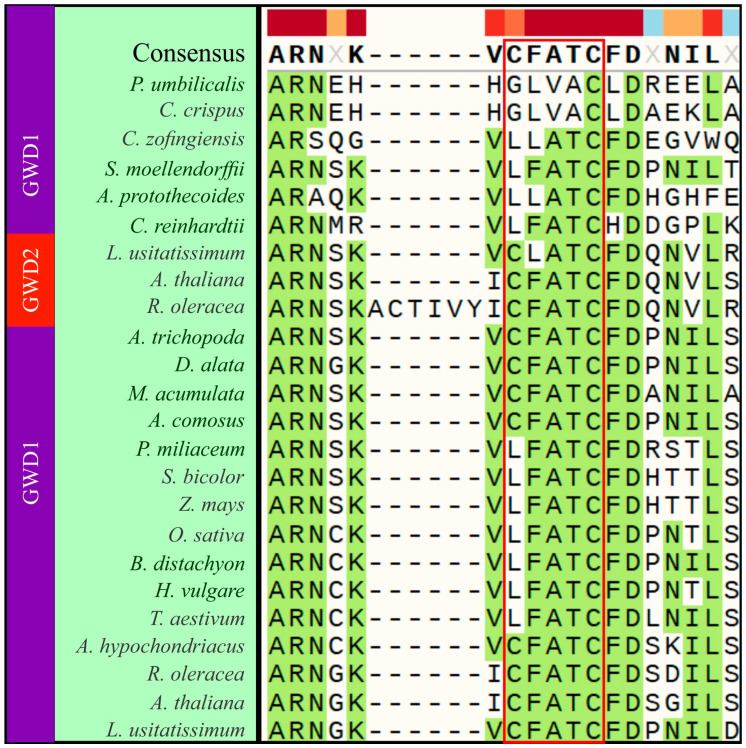
Substitution of amino acid residues in some plants at redox regulation motif (CFACT).

The existence of two tandem starch binding domains (SBD), known as carbohydrate binding module 45 (CBM45), on CaZy database [43], was previously reported [44,45]. We examined the presence of this motif, with emphasis on the five aromatic amino acid residues crucial for its function. In most of the phyla, the five aromatic amino acid residues were conserved in the two CBM45 tandems, designated CBM45-1 (Figure 2a) and CBM45-2 (Figure 2b). However, some deletions and substitutions were observed. For instance, we found a complete deletion in the GWD1 of *C. zofingiensis* and *G. hirsutum*, and partial deletions in *A. protothecoides*, *C. rabica*, *P. miliaceum*, and the GWD2 of *M. esculenta*. The five aromatic amino acid residues were mostly conserved in CBM45-2 of the phyla regardless of protein subgroup (GWD1 or GWD2), except in *T. cacao* and *P. miliaceum*. Contrarily, almost all the five aromatic amino acids were conserved in CBM45-2 of *A. protothecoides*, except the second phenylalanine which was substituted by tyrosine (Y). Furthermore, we observed that the CFACT motif linked to redox regulation of GWD was present and conserved in most of the plants except in rhodophytes, lycophytes, most of the chlorophytes (Figure 3), and mostly in grasses (monocots).

### 3.5. Analysis of Amino Acid Mutation

The potato GWD sequence was used as the reference sequence in PROVEAN to predict whether the variant effect in plants where substitution of conserved amino acids and redox regulation motifs were observed is deleterious or neutral. We observed a total of 55 amino acid variants; 37 for the conserved tandem domains and 18 for the predicted redox regulation motifs. In the tandem domains, neutral mutation (F substituted with Y) was predicted only in one position for each of *A. protothecoides* and *P. patens* GWDa and b, *M. polymorpha* and *S. magellanicum* (Table 3). Deleterious mutation was predicted for the remaining 33 variants in the conserved tandem domains of the following plants; *C. crispus*, *C. zofingiensis*, *G. hirsutum* (GWD1), *A. protothecoides*, *P. miliaceum*, *C. arabica*, *M. esculenta* (GWD2), *C. reinhardti* and *P. umbilicalis*. Contrarily, mutations at redox regulation motif were neutral in most of the positions, except seven positions, involving three positions for *C. crispus* (C1079G, F1080L and A1081V), *L. usitatissimum*/GWD2 (F1080L) and *P. umbilicalis* (C1079G, F1080L and A1081V).

## 4. Discussion

We examined alpha-water dikinase (GWD) sequences to elucidate its evolution in five plant phyla utilizing various computational approaches. Our phylogenetic tree reveals that the GWD enzyme may have evolved different protein sequences, while maintaining core function of the ancestral gene. This might be due to the fact that the selection pressure that led to the evolution of these sequences ensured that ancestral enzyme function is maintained in descendant species [46]. Two copies of the GWD gene were also observed in some angiosperm families, suggesting a divergence in function between GWD1 and GWD2 paralogs of these species. A different functional evolution may exist regarding PWD, another paralog of GWD, as described [2], but not the focus of current study. In *M. esculenta*, GWD2 and the other GWD isoforms were implicated by Zhou et al. [45] in storage starch degradation, due to increased expressions during post-harvest physiological deterioration where its activity was linked to seed development in *A. thaliana* and not the degradation of transitory starch at night [8]. However, gain of GWD2 in several plant families points to another unique event during GWD evolution. A similar occurrence has been reported in some other plants with an isoform of starch synthase (SSIV) and starch branching enzyme (SBEI) in *A. thaliana* and *B. rapa*, and a third isoform of starch phosphorylase (PHO3) [47,48,49]. Our result demonstrates that GWD1 sequences of rhodophytes are basal first from the rest of the phyla, indicating it is the most ancestral compared to other phyla.

Evolutionary events like amino acid substitutions, deletions, insertions, domain and gene duplications observed in the MSA suggest that GWD enzymatic activities may have been further modified post-speciation, possibly in response to environmental selection pressure. A recent report showed that these changes are capable of causing minor functional (creeping evolution) or drastic change [16]. Out of the nine cysteine residues present in potato GWD, only two were experimentally shown to form reversible disulfide linkage [39], which activate and inactivate GWD. While it is unclear why the number of cysteine residues is remarkably higher in *C. reinhardtii* or all the GWD2 protein sub-groups, we hypothesize that the occurrence of more cysteine may not only contribute to the stability of its GWD protein structure, but may increase its ability to form more disulfide connections with other cellular molecules. In addition, abundance of conserved glycine residues at various positions in the C-terminus, possibly implies that this amino acid is functionally important [9].

The aliphatic index was generally high for all the plants, indicating GWD’s thermal stability, potentially a requirement for maintaining a periodical soluble form. According to Ritte et al. [50], GWD partly exist in a soluble form in illuminated leaves, and thermostability is correlated with protein solubility [51]. While other factors such as protein aggregation propensity and folding rate may also contribute to solubility [52], high thermostability may prevent GWD structural decomposition during photosynthesis.

We detected a predicted localization signal in almost all angiosperms but almost none in lower plants, implying that during evolution, GWD1 gained the ability to be chloroplast localized from its ancestral state and it has been maintained. However, we found no signal sequence in GWD2, possibly a deletion for novel localization-based function in those plants. However, the enzyme may be transited by through complexing with other proteins targeting plastids or mitochondria. Both cTP and mTP had been postulated to share similar evolutionary mechanisms [53] and their current functions were suggested to emanate from selection pressure, leading to shuffling and streamlining of separate exons to form multiple domains. This may have been responsible for the high variation we observed in the predicted amino acid sequences, lengths and arrangement, as reported [54,55]. This location at the N-terminus in MSA was also the least conserved region, giving an indication of the intense selection pressure in the evolution of the current function of transit peptides. The absence of transit peptides in GWD2 in the plant species in which it is present supports the fact that they are localized in the cytosol [8]. Finally, recent studies have established that cTP and mTP contain specific motifs [56] and amino acid residues [57,58], with which they either interact with cytosolic complexes containing Hsp70 and Hsp90 (molecular chaperones that target transit peptides and guide the preprotein to the outer membranes of the organelles) or TOC–TIC (chloroplast)/TOM–TIM (mitochondria) protein import machinery [59,60]. Proline has been reported to play a crucial role in efficient protein translocation into the chloroplast through import channels. However, proline was not conserved at any common location in the predicted signal sequences, though it was abundant in all the plants whose GWD were predicted to bear cTP. Hence, our prediction might be helpful in identifying those motifs embedded in the respective transit peptides of some of the plants and such knowledge may be useful in elucidating GWD transit to localized sites in plants.

The conversion of starch into soluble sugar, a process known as amylolysis, is an essential process in planta. However, the semi-crystalline structure of starch poses a limitation to enzymes involved in its breakdown. For efficient amylolysis, such enzymes have evolved substrate-binding sites on the catalytic module [61] or specialized carbohydrate-binding-module (CBM), known as starch-binding domain (SBD) [62]. CBM is an auxiliary module (classified into several families) of about 40 to 200 amino acids with a distinctive fold with which it binds to carbohydrate and adjacent to the carbohydrate-active enzyme [63]. It exists as two tandem domains in most of the plants studied and a similar domain arrangement was found in the plastidial α-amylase AMY3 in *A. thaliana* (separated by 50 linker amino acids), whose functional role as starch (CBM) was inferred from knock-out studies involving phosphoglucan phosphatase (SEX4) [64]. The two modules were suggested to arise as a result of gene duplication (Mikkelsen et al., 2006), and may have led to alterations in the functionality of the conjoining catalytic domain [16]. For example, in a transgenic potato line in which CBM45-1 was missing, the shorter glucan chains were preferred substrates for phosphorylation [43]. However, the stored starch in the endosperm of *Z. mays* and *H. vulgare* has been reported to have negligible phosphate content, even though the two CBM45 were fully present in GWD sequences of both plants. We suspect that for both CBM45 tandem domains in *Z. mays* and *H. vulgare*, degenerative mutations may have occurred within the domains, leading to altered enzyme structure and function. However, the five aromatic amino acid residues in CBM45s were conserved across most of the plants and various mutations in these residues were highly deleterious. Mutation of these conserved residues may be due to a more intense selection pressure. These mutations may affect glucan substrate specificity or alter its folding or function. It is unclear how GWD1 enzyme in *G. hirsutum* and *C. zofingiensis* successfully perform its function because the CBM45-1 tandem was completely absent, and their absence was predicted to be deleterious. We surmise that this enzyme may have evolved effective use of CBM45-2 in performing its function, or it may form complexes with other proteins, thereby providing a robust alternative to the deleterious effects of the mutation. Predicted deleterious mutations may affect the starch binding ability of GWD. For plants in which mutations at GWD redox motif may have occurred, they may utilize another cysteine in the sequence or devise another means by which it redox regulate the enzyme.

## 5. Conclusions

In this study, we used various computational approaches to compare the GWD sequences of 48 plant species, with our results providing an insight into the evolutionary variation in GWD catalytic activity among plants. Deleterious mutations were identified for some plants at various positions of the five aromatic amino acids, which are highly conserved in tandems of CBM45 and vital for binding of the enzymes to starch. These mutations may be responsible for altered carbohydrate binding activity of GWD in plants, thereby affecting phosphorylation of transit and stored starch. This study has the potential to guide ongoing and future efforts towards producing modified starch in plants, targeted at reducing environmental pollution occasioned by the artificial modification of industrial starch phosphate content. Finally, this study has provided a broader understanding of the complexity involved in the evolution of GWD enzymes in various plants during speciation.

## 6. Declaration of Competing Interest

Authors declare we have no competing financial or personal interest.

## Figures and Tables

**Table 1:** Physio–chemical properties of the GWD protein in selected plant species.

Species	Protein	Size	MW	II	AI	GRAVY	Cysteines	DC Prediction
*P. umbilicalis*	GWD1	1343	144,770.79	38.61	88.75	−0.172	18	9
*C. crispus*	GWD1	1353	150,301.30	39.12	88.14	−0.343	15	7
*C. zofingiensis*	GWD1	1086	118,300.07	45.26	87.13	−0.120	18	9
*A. protothecoides*	GWD1	1252	135,517.80	34.80	94.46	−0.122	10	5
*C. reinhardtii*	GWD1	1411	154,283.18	39.07	86.88	−0.282	24	12
*S. moellendorffi*	GWD1	1309	146,481.65	39.89	92.80	−0.330	10	5
*M. polymorpha*	GWD1	1424	156,626.81	44.27	89.37	−0.336	14	7
*S. magellanicum*	GWD1	1546	170,633.58	43.31	91.60	−0.294	18	9
*P. patens GWDa*	GWD1	1415	157,825.51	43.24	93.82	−0.284	13	6
*P. patens GWDb*	GWD1	1420	157,172.42	37.61	88.67	−0.336	14	7
*A.trichopoda*	GWD2	1302	146,933.48	43.48	95.38	−0.303	21	10
*L. usitatissimum*	GWD2	1325	149,512.75	44.00	96.78	−0.250	19	9
*M. esculenta*	GWD2	1228	138,500.88	38.41	95.01	−0.239	20	10
*R. communis*	GWD2	1300	146,401.26	40.44	94.83	−0.205	27	13
*G. hirsutum*	GWD2	1303	146,156.91	41.26	96.57	−0.192	23	11
*T. cacao*	GWD2	1246	140,321.26	38.25	92.54	−0.235	24	12
*C. clementina*	GWD2	1244	141,040.53	44.60	93.63	−0.253	25	12
*M. maritima*	GWD2	1276	144,304.40	41.83	92.79	−0.294	28	14
*C. rubella*	GWD2	1278	144,939.00	41.05	92.34	−0.302	20	10
*A. thaliana*	GWD2	1278	144,811.75	43.11	92.34	−0.308	22	11
*M. perfoliatum*	GWD2	1243	140,157.32	38.33	91.65	−0.287	25	12
*B. oleracea*	GWD2	1400	158,307.56	41.00	91.60	−0.278	31	15
*B. rapa*	GWD2	1281	144,354.66	38.83	92.73	−0.264	27	13
*A. trichopoda*	GWD1	1385	155,862.42	45.67	91.28	−0.400	10	5
*D. alata*	GWD1	1515	169,131.63	43.87	89.46	−0.374	11	5
*M. acumulata*	GWD1	1611	181,353.26	46.94	92.58	−0.331	15	7
*A. comosus*	GWD1	1474	165,093.79	45.80	93.32	−0.332	14	7
*P. miliaceum*	GWD1	1468	163,391.24	47.62	91.39	−0.343	11	5
*S. bicolor*	GWD1	1469	164,264.97	47.06	88.90	−0.385	10	5
*Z. mays*	GWD1	1469	163,962.14	47.30	87.71	−0.394	9	4
*O. sativa*	GWD1	1203	134,156.82	43.91	89.34	−0.364	11	5
*B. distachyon*	GWD1	1455	162,600.00	46.05	90.10	−0.349	11	5
*H. vulgare*	GWD1	1410	157,590.85	42.25	88.57	−0.375	9	4
*T. aestivum*	GWD1	1374	154,082.86	42.83	89.68	−0.372	9	4
*A. hypochondriacus*	GWD1	1398	156,105.95	44.12	89.88	−0.380	12	6
*H. annuus*	GWD1	1456	163,484.38	41.30	90.36	−0.394	13	6
*C. arabica*	GWD1	1474	165,508.56	48.40	88.06	−0.439	11	5
*N. tabacum*	GWD1	1464	163,426.17	44.00	90.78	−0.370	10	5
*C. annuum*	GWD1	1464	163,598.62	40.00	91.78	−0.351	9	4
*S. lycopersicum*	GWD1	1465	163,791.47	41.29	91.31	−0.356	11	5
*S. chacoense*	GWD1	1432	159,732.07	40.65	91.85	−0.351	10	5
*S. tuberosum*	GWD1	1463	163,261.11	40.90	92.05	−0.351	10	5
*V. vinifera*	GWD1	1478	165,452.70	39.46	91.79	−0.353	12	6
*F. vesca*	GWD1	1400	155,592.13	41.59	91.84	−0.359	13	6
*M. domestica*	GWD1	1405	156,915.34	39.52	87.72	−0.426	8	4
*C. melo*	GWD1	1471	164,902.74	42.29	93.06	−0.370	10	5
*G. max*	GWD1	1459	163,761.56	40.49	89.80	−0.406	10	5
*P. vulgaris*	GWD1	1456	163,484.38	41.30	90.36	−0.394	13	6
*V. unguiculata*	GWD1	1455	163,535.52	41.22	91.19	−0.386	11	5
*L. usitatissimum*	GWD1	1480	164,831.39	46.33	87.55	−0.384	12	6
*M. esuculenta*	GWD1	1409	157,838.14	45.03	91.34	−0.346	9	4
*R. communis*	GWD1	1469	164,321.24	46.37	90.13	−0.388	9	4
*G. hirsutum*	GWD1	1471	164,719.18	42.70	90.74	−0.399	12	6
*T. cacao*	GWD1	1470	164,632.01	42.34	89.82	−0.386	12	6
*C. clementina*	GWD1	1388	155,729.81	44.94	91.97	−0.361	11	5
*C. papaya*	GWD1	1473	165,710.38	40.31	92.52	−0.391	11	5
*M. perfoliatum*	GWD1	1413	158,428.12	41.53	89.89	−0.407	10	5
*B. oleracea*	GWD1	1396	156,676.20	44.73	89.16	−0.428	11	5
*B. rapa*	GWD1	1399	156,557.78	42.31	90.09	−0.390	11	5
*M. maritima*	GWD1	1401	156,892.19	42.20	90.31	−0.401	10	5
*C. rubella*	GWD1	1392	155,931.95	44.29	89.29	−0.429	10	5
*A. thaliana*	GWD1	1399	156,581.78	41.14	89.74	−0.414	10	5

DC: disulphide connectivity; MW: molecular weight; II: instability index; AI: aliphatic index; GRAVY: grand average of hydropathicity.

**Table 2:** Prediction of GWD localization site.

Species	Protein	TargetP 2.0						Multiloc2		
		cTP	mTP	Others	Loc	TPlen	Ch	Cy	M	SP
*P. umbilicalis*	GWD1	0.00	0.00	1.00	_		0.54	0.29	0.08	0.00
*C. crispus*	GWD1	0.00	0.00	1.00	_		0.21	0.72	0.03	0.00
*C. zofingiensis*	GWD1	0.13	0.00	0.87	_		0.11	0.70	0.01	0.16
*A. protothecoides*	GWD1	0.00	0.00	0.99			0.28	0.65	0.04	0.00
*C. reinhardtii*	GWD1	0.78	0.19	0.06	C	75–76	0.96	0.02	0.02	0.0
*S. moellendorffi*	GWD1	0.00	0.00	1.00	_		0.48	0.06	0.45	0.01
*M. polymorpha*	GWD1	0.00	0.00	1.00	_		0.84	0.07	0.06	0.00
*S. magellanicum*	GWD1	0.02	0.00	0.98	_		0.89	0.04	0.06	0.00
*P. patens GWDa*	GWD1	0.00	0.40	0.53	_		0.87	0.05	0.06	0.00
*P. patens GWDb*	GWD1	0.27	0.47	0.26	M	68–69	0.60	0.02	0.35	0.00
*A. trichopoda*	GWD2	0.00	0.00	1.00	_		0.73	0.19	0.03	0.00
*L. usitatissimum*	GWD2	0.00	0.00	0.98	_		0.06	0.28	0.02	0.63
*M. esculenta*	GWD2	0.01	0.00	0.99	_		0.32	0.50	0.04	0.12
*R. communis*	GWD2	0.00	0.00	0.99	_		0.34	0.51	0.04	0.00
*G. hirsutum*	GWD2	0.02	0.o1	0.95	_		0.64	0.24	0.09	0.00
*T. cacao*	GWD2	0.00	0.00	0.99	_		0.70	0.17	0.09	0.01
*C. clementina*	GWD2	0.00	0.00	1.00	_		0.28	0.38	0.04	0.24
*M. maritima*	GWD2	0.01	0.12	0.87	_		0.71	0.17	0.08	0.00
*C. rubella*	GWD2	0.00	0.00	1.00	_		0.12	0.6	0.07	0.00
*A. thaliana*	GWD2	0.00	0.00	1.00	_		0.15	0.67	0.13	0.00
*M. perfoliatum*	GWD2	0.00	0.00	1.00	_		0.13	0.6	0.07	0.00
*B. oleracea*	GWD2	0.00	0.00	1.00	_		0.09	0.33	0.11	0.03
*B. rapa*	GWD2	0.00	0.05	0.93	_		0.19	0.59	0.14	0.00
*A. trichopoda*	GWD1	0.00	0.00	1.00	_		0.16	0.76	0.03	0.00
*D. alata*	GWD1	0.71	0.00	0.28	C	96–97	0.70	0.19	0.07	0.00
*M. acumulata*	GWD1	0.07	0.00	0.79	_		0.80	0.10	0.07	0.02
*A. comosus*	GWD1	0.91	0.00	0.08	C	85–86	0.94	0.01	0.04	0.00
*P. miliaceum*	GWD1	0.97	0.00	0.03	C	61–62	0.95	0.02	0.03	0.00
*S. bicolor*	GWD1	0.99	0.00	0.00	C	65–66	0.94	0.03	0.03	0.00
*Z. mays*	GWD1	0.97	0.00	0.03	C	65–66	0.94	0.03	0.03	0.00
*O. sativa*	GWD1	0.96	0.01	0.03	C	62–63	0.90	0.01	0.04	0.00
*B. distachyon*	GWD1	0.96	0.00	0.04	C	57–58	0.94	0.01	0.05	0.00
*H. vulgare*	GWD1	0.03	0.59	0.39	M	16–17	0.47	0.04	0.49	0.00
*T. aestivum*	GWD1	0.00	0.00	1.00	_		0.48	0.37	0.1	0.01
*A. hypochondriacus*	GWD1	0.00	0.05	0.95	_		0.70	0.16	0.02	0.00
*H. annuus*	GWD1	0.91	0.02	0.07	C	81–82	0.92	0.01	0.02	0.00
*C. arabica*	GWD1	0.00	0.00	1.0	_		0.74	0.02	0.23	0.00
*N. tabacum*	GWD1	0.81	0.03	0.16	C	76–77	0.97	0.01	0.02	0.00
*C. annuum*	GWD1	0.90	0.02	0.07	C	76–77	0.44	0.50	0.03	0.00
*S. lycopersicum*	GWD1	0.76	0.02	0.21	C	77–78	0.94	0.03	0.03	0.00
*S. chacoense*	GWD1	0.81	0.04	0.16	C	76–77	0.96	0.01	0.02	0.00
*S. tuberosum*	GWD1	0.76	0.03	0.2	C	76–77	0.96	0.02	0.01	0.00
*V. vinifera*	GWD1	0.51	0.26	0.23	C	79–80	0.91	0.03	0.05	0.00
*F. vesca*	GWD1	1.00	0.00	0.00	C	69–70	0.94	0.01	0.04	0.00
*M. domestica*	GWD1	0.93	0.00	0.06	C	76–77	0.94	0.01	0.04	0.00
*C. melo*	GWD1	0.61	0.11	0.28	C	84–85	0.92	0.01	0.07	0.00
*G. max*	GWD1	0.68	0.01	0.24	C	72–73	0.95	0.01	0.04	0.00
*P. vulgaris*	GWD1	0.74	0.00	0.16	C	54–55	0.89	0.05	0.04	0.01
*V. unguiculata*	GWD1	0.50	0.00	0.4786	C	60–61	0.95	0.07	0.02	0.00
*L. usitatissimum*	GWD1	0.80	0.00	0.19	C	91–92	0.96	0.01	0.03	0.00
*M. esuculenta*	GWD1	0.88	0.02	0.10	C	83–84	0.93	0.04	0.03	0.00
*R. communis*	GWD1	0.66	0.04	0.29	C	82–83	0.92	0.03	0.04	0.00
*G. hirsutum*	GWD1	0.77	0.01	0.23	C	84–85	0.94	0.01	0.05	0.00
*T. cacao*	GWD1	0.90	0.00	0.10	C	84–85	0.87	0.05	0.06	0.00
*C. clementina*	GWD1	0.51	0.08	0.41	C	91–92	0.28	0.38	0.04	0.00
*C. papaya*	GWD1	0.01	0.00	0.99	_		0.93	0.01	0.05	0.00
*M. perfoliatum*	GWD1	0.26	0.02	0.72	_		0.91	0.01	0.05	0.00
*B. oleracea*	GWD1	0.81	0.10	0.10	C	74–75	0.77	0.05	0.15	0.01
*B. rapa*	GWD1	0.42	0.40	0.17	C	75–76	0.84	0.01	0.11	0.00
*M. maritima*	GWD1	0.37	0.54	0.08	M	40–41	0.91	0.01	0.03	0.00
*C. rubella*	GWD1	0.58	0.22	0.20	C	67–68	0.85	0.05	0.08	0.00
*A. thaliana*	GWD1	0.53	0.22	0.25	C	74–75	0.80	0.08	0.11	0.01

Localization site (Loc); (C/Ch) chloroplast, i.e., sequence contains chloroplast transit peptide cTP; (M) Mitochondrion i.e., sequence contains mitochondrial targeting peptide mPT, (Cy) cytoplasm and (S) Secretory pathway (SP), i.e., sequence contains, a signal peptide and (_) means other location. The predicted location is that with the highest value among the possible locations. Reliability class (RC) ranges between 1 to 5, where 1 indicates the strongest prediction and the lower the value of RC the safer the prediction. The value of Tplen indicates the predicted presequence length.

**Table 3:** Prediction of mutational effects on GWD of selected plants.

Species	CBM45−1		CBM45−2		CFACT	
	Position	Score	Position	Score	Position	Score
Irish moss	W139R	−5.506	F520A	−5.14	C1079G	−5.57
			F536L	−3.65	F1080L	−4.20
					A1081V	−3.70
*C. zofingiensis*	W129del	−11.64			C1079L	0.27
	W139del	−9.03				
	F184del	−7.88				
	W194del	−10.49				
	F202del	−7.56				
*G. hirsutum*	W129del	−11.64				
	W139del	−9.03				
	F184del	−7.88				
	W194del	−10.49				
	F202del	−7.56				
*A. protothecoides*	W129L	−7.45	F536Y	−1.18	C1079L	0.27
	W139T	−5.82				
	F184T	−4.62				
	W194del	−10.49				
	F202E	−2.95				
*P. miliaceum*	W194C	−7.55	F520del	−8.40	C1079L	0.27
	F202del	−7.56	W528del	−12.34		
			F536del	−7.40		
*C. arabica*	W129del	−11.64				
*M. esculenta*/GWD2	W129del	−11.06				
*C. reinhardtii*	W129R	−8.04			C1079L	0.27
	W139L	−6.078				
*S. bicolor*					C1079L	0.27
*Z. mays*					C1079L	0.27
*O. sativa*					C1079L	0.27
*B. distachyon*					C1079L	0.27
*H. vulgare*					C1079L	0.27
*T. aestivum*					C1079L	0.27
*P. umbilicalis*	W139S	−5.93	F520A	−5.14	C1079G	−5.57
			F536M	−3.72	F1080L	−4.20
					A1081V	−3.70
*S. moellendorffi*					C1079L	0.27
*M. polymorpha*			F536Y	−1.18		
*S. magellanicum*			F536Y	−1.18		
*P. patens* (GWDA and B)			F536Y	−1.18		
*T. cacao*			F520del	−8.40		
			W528del	−12.34		
			F536del	−7.40		

CBM-carbohydrate binding module; red color represents deleterious mutation; black color is neutral mutation; values lower than −2.5 are considered deleterious; values above −2.5 are considered neutral.

## Supplementary Materials

The following are available online at https://www.mdpi.com/2223-7747/9/9/1101/s1, Table S1: Accession numbers of complete GWD amino acid sequences for plant species.
