# Supplementary material for: Differential Evolution of α-Glucan Water Dikinase (GWD) in Plants
Source: Plants (Basel). 2020 Aug 27;9(9):1101. doi: 10.3390/plants9091101 (PMC7569903; doi:10.3390/plants9091101)
Supplement: Supplementary file 1 [file plants-09-01101-s001.pdf]

Table S1. Accession numbers of complete GWD amino acid sequences for the plant species.

S/No	Species	UniProt ID	GenBank Accession #	Phytozome ID
1	*P. umbilicalis*			Pum1598s0001.1.p
2	*C. crispus*	R7QKK2		
3	*C. zofingiensis*			Cz09g00250.t1
4	*A. protothecoides*	A0A087SJ57		
5	*C. reinhardtii*	A0A2K3DIY0		
6	*S. moellendorffi*			266998
7	*M. polymorpha*	A0A2R6X3K3		
8	*S. magellanicum*			Sphmag17G074900.1
9	*P. patens GWDa*			Pp3c3_11200V3.3.p
10	*P. patens GWDb*			Pp3c8_6536V3.3.p
11	*A.trichopoda*			AmTr_v1.0_scaffold0003 3.183
12	*L. usitatissimum*			Lus10032829
13	*M. esculenta*		AFO83530.1	
14	*R. communis*		XP_015579774.1	
15	*G. hirsutum*			Gohir.A05G216400.2
16	*T. cacao*	A0A061FDU7		
17	*C. clementina*			Ciclev10017434m
18	*M. maritima*			Mamar.0039s0196.1
19	*C. rubella*			Carub.0007s1662.1.p
20	*A. thaliana*	Q9STV0		
21	*M. perfoliatum*			Myper.0039s1058.1.p
22	*B. oleracea*			Bol009541
23	*B. rapa*			Brara.A01450.1.p
24	*A. trichopoda*	W1P456		
25	*D. alata*			Dioal.17G119100.2.p
26	*M. acumulata*	M0SEL4_MUSAM		
27	*A. comosus*	A0A199UE45		
28	*P. miliaceum*	A0A3L6S324		
29	*S. bicolor*	C5Z316		
30	*Z. mays*	A0A1D6LTL9		
31	*O. sativa*		XM_015787980.2	
32	*B. distachyon*			BdiBd21-3.1G0542600.2
33	*H. vulgare*			Hr1G048330.1
34	*T. aestivum*			Traes_7AS_0F7738049.1
35	*A. hypochondriacus*			AH004443-RA
36	*H. annuus*	A0A251T3N7		
37	*C. Arabica*			Scaffold_608.124
38	*N. tabacum*	A0A1S3YFK2		
39	*C. annuum*	A0A2G2YEX8		
40	*S. lycopersicum*	B5B3R3		
41	*S. chacoense*	A0A0V0IZQ3		
42	*S. tuberosum*	Q9AWA5		
43	*V. vinifera*	D7TDL2		
44	*F. vesca*			gene03581
45	*M. domestica*			MD16G1097800
46	*C. melo*	A0A1S3BEF3		

47	*G. max*	I1KXC2	
48	*P. vulgaris*	V7C6L3	
49	*V. unguiculata*		VuTZ30.03G211100.1.p
50	*L. usitatissimum*		Lus10013044
51	*M. esuculenta*	V9K755	
51	*R. communis*	B9SPI3	
53	*G. hirsutum*		Gohir.A12G144400.1.p
54	*T. cacao*		Thecc.02G256300.4.p
55	*C. clementina*		Ciclev10030521m
56	*C. papaya*		evm.model.supercontig_ 69.90
57	*M. perfoliatum*		Myper.0016s0127.1.p
58	*B. oleracea*	AOAOD3DNZ7	Bol031282
59	*B. rapa*		Brara.I05187.1.p
60	*M. maritima*		Bol031282
61	*C. rubella*		Carub.0001s1031.1.p
62	*A. thaliana*	Q9SAC6	
